# Inequalities in cancer diagnostic outcomes for patients with a learning disability: a retrospective cohort study in England

**DOI:** 10.1136/bmjonc-2026-001104

**Published:** 2026-05-18

**Authors:** Bianca Wiering, Gary A Abel, Leon Farmer, Robert Kerrison, Samuel W D Merriel, Sarah Price, David Shotter, Jose M Valderas, Luke T A Mounce

**Affiliations:** 1 Department of Health and Community Sciences, University of Exeter, Exeter, UK; 2 University of Surrey, Guildford, UK; 3 Centre for Primary Care and Health Services Research, The University of Manchester, Manchester, UK; 4 National University of Singapore, Singapore

**Keywords:** Mortality, Epidemiology, Health Services Research

## Abstract

**Objectives:**

The cancer diagnostic process may be more complicated for patients with a learning disability (‘intellectual disability’ outside of the UK) than for other patients. We aimed to investigate whether patients with a learning disability were more likely to experience disadvantage in cancer diagnostic pathways and outcomes.

**Design:**

A retrospective cohort study using routinely collected linked data across primary care and cancer registration.

**Setting:**

1470 general practices in England.

**Participants:**

277 050 participants who were aged 40+ years, had an incident cancer recorded in the cancer registration during 2012–2018 and were registered for at least 3 years at their general practice prior to diagnosis.

**Main outcome measures:**

Emergency presentation route to diagnosis or urgent suspected cancer referral route to diagnosis, cancer stage at diagnosis (early vs advanced) and all-cause mortality within 30 days after diagnosis.

**Results:**

277 050 patients were included in the overall study, of which 211 423 were included in analyses with cancer stage as outcome. Learning disability was recorded for 796 (0.3%) patients. Patients with a learning disability were over twice as likely to be diagnosed via an emergency presentation (adjusted OR (aOR): 2.65, 95% CI 2.26 to 3.11, p<0.001) and half as likely to be diagnosed via an urgent suspected cancer referral (aOR: 0.51, 95% CI 0.43 to 0.60, p<0.001). They were also more likely to be diagnosed with advanced-stage cancer (aOR: 1.37, 95% CI 1.12 to 1.67, p=0.002), especially for breast cancer, and to die within 30 days of diagnosis (aOR: 3.77, 95% CI 3.10 to 4.59, p<0.001) than patients without a learning disability.

**Conclusions:**

Patients with a learning disability experience marked inequalities in cancer diagnostic pathways and outcomes. The increased risk of advanced-stage breast cancer is of particular note. Improved support to access and navigate the health care system may be required to negate experienced difficulties during the diagnostic process.

**WHAT IS ALREADY KNOWN ON THIS TOPIC**

- Patients with a learning disability experience avoidable mortality after a cancer diagnosis.
- Greater dependence on others, low symptom awareness and difficulties accessing and navigating the healthcare system during the cancer diagnostic process potentially contribute to worse outcomes, particularly for more severe learning disability.

**WHAT THIS STUDY ADDS**

- Considering all cancer sites except non-melanoma skin cancer, we found that having a record of a learning disability was associated with an increased likelihood of being diagnosed at an advanced stage, especially for breast cancer, and increased mortality within 30 days after diagnosis.
- Patients with a learning disability were more likely to be diagnosed as an emergency, and less likely to be diagnosed via an urgent suspected cancer referral.

**HOW THIS STUDY MIGHT AFFECT RESEARCH, PRACTICE OR POLICY**

- Patients with a learning disability require better support to negate existing inequalities in diagnostic pathways and outcomes.

## Introduction

 Cancer survival rates have improved greatly in recent decades.^1^,^3^ Some patient groups, however, such as patients with a learning disability–synonymous with ‘intellectual disability’, as preferred outside of the UK,^4^ still experience significant avoidable cancer mortality.^5^ A learning disability is defined as a lifelong impaired intellectual ability, and reduced social functioning. It covers varying levels of intellectual ability, experienced difficulties and support needs and is estimated to affect around 1–3% of the population worldwide.^6^ Improving care for patients with a learning disability is a priority for the English National Health Service (NHS), and is included in the NHS long-term plan,^7^ the Health and Social Care Act 2022,^8^ and the Core20PLUS5 approach to tackling healthcare inequalities.^9^ These efforts are very timely, as an English report on the lives and deaths of patients with a learning disability found that cancer was the second most common cause of death and accounted for 15.7% of avoidable deaths.^10^

A potential reason for avoidable cancer mortality may be found in delayed diagnosis. Patient outcomes tend to be better if patients are diagnosed early, as it increases the number of available treatment options and their success, while reducing treatment length, complications and long-term cancer and treatment impacts.^11 12^ People with a learning disability, however, experience difficulties in recognising possible cancer symptoms,^13^ communicating their experiences and accessing and navigating the healthcare system.^14^,^16^ A recent review suggested that people with a learning disability, their carers and healthcare practitioners have low awareness of possible cancer symptoms, and that carers and healthcare professionals often were not sure of their role in facilitating cancer awareness for patients with a learning disability.^13^ Ethnographic studies found that people with a learning disability may have higher levels of dependence on others to recognise, acknowledge and explain possible cancer symptoms and help them access and navigate the healthcare system. Additionally, carers often have an important role in what information is shared, and in deciding whether a person with a learning disability is able to cope with the cancer diagnostic process and subsequent treatments.^15 17^

These factors are likely to complicate the diagnostic process, and could contribute to a delayed diagnosis.^15^ Our National Institute for Health and Care Research (NIHR) funded programme (SPOtting Cancer among Comorbidities (SPOCC), NIHR201070) explored cancer diagnostic processes and outcomes in patients with 121 pre-existing health conditions. Here, we present findings relating to the following research questions:

1. Are patients with a learning disability more likely to experience suboptimal pathways to diagnosis than patients without a learning disability (ie, increased emergency presentations and decreased urgent suspected cancer referrals in primary care)?
2. Are patients with a learning disability more likely to be diagnosed with advanced-stage cancer than patients without a learning disability?
3. Are patients with a learning disability more likely to die within 30 days of their cancer diagnosis than patients without a learning disability?

## Materials and methods

### Data source

The study is a retrospective cohort study using linked Clinical Practice Research Datalink (CPRD) data. CPRD is an electronic database of routinely collected de-identified primary care health records in the UK. For this study, CPRD Aurum data relating to English patients was used. CPRD Aurum covered 10% of general practitioner (GP) practices in England in 2018.^18^ This data was linked to the National Cancer Registration and Analysis Service (NCRAS),^19^ the patient-level Index of Multiple Deprivation (IMD) 2015 and National Office for Statistics death data. Linkage was carried out by a trusted third party at NHS England. No patient-identifiable data are received by researchers using the linked data.^20^ CPRD linked datasets are validated datasets, which have widely been used for cancer diagnostic studies.^21^,^25^

### Study population

The initial number of patients included in the study population was subject to linked data availability in CPRD. We included all patients aged 40 years and older, with primary care data available in CPRD, an incident cancer diagnosis recorded in NCRAS data between 01 January 2012 and 31 December 2018, and 3 years continued registration at their practice before their cancer diagnosis. The inclusion period lower boundary was chosen to optimise record completeness, while the upper boundary was based on the availability of NCRAS linkage at the time of the study. Patients were excluded if they: (1) had a recorded sex not matching a sex-specific cancer (eg, male patients with uterine cancer); (2) had a cancer diagnosis recorded before the inclusion time period; (3) were diagnosed via screening; (4) were diagnosed with non-melanoma skin cancer; (5) died before their diagnosis (online supplemental figure 1).

### Patient and public involvement

The study is embedded in a Programme Grant, which was designed with extensive patient and public involvement (PPI) consultation. The programme has a patient co-investigator, and a PPI group, with PPI members fulfilling bridge roles for work packages.^26^ For this study, the PPI bridge (LF) attended three work package meetings and contributed to the manuscript. One meeting was held with the PPI group to disseminate results.

### Patient characteristics

Patient age, sex (male/female) and smoking history (never/ever) were extracted from CPRD. Age was treated as a categorical variable with 5-year age groups between 40 and 89 years and a 90 years and older group. IMD groups were defined based on national quintiles. The Cambridge Multimorbidity Score (CMS) general outcome weighting^27^ was used to measure morbidity burden at least a year before diagnosis. The CMS is a validated measure consisting of 37 long-term conditions, and is weighted by primary care use, mortality and unplanned hospital admissions.^27^ Where available, pre-existing validated code lists were used to capture CMS conditions. New code lists were developed using robust methods.^28^ Four morbidity burden groups (none, low, medium, high) were created, with the last three based on CMS tertiles excluding the weighting for learning disability.

### Learning disability

Learning disability was measured using definitions developed for the CMS.^27^ A validated code list developed for the Quality and Outcomes Framework (business rules V.45) was used.^29^ The Quality and Outcomes Framework is a pay-for-performance scheme in the UK that rewards GP practices based on indicators, one of which is to maintain a register of patients with a learning disability.^30^ Patients were categorised as having a learning disability if a learning disability code was recorded at least 12 months before their cancer diagnosis.

### Cancer site and stage

Cancer diagnoses and stage were extracted from NCRAS, and the tumour with the earliest diagnosis date was included. In rare cases where multiple diagnoses were recorded on the same date, the most advanced-stage cancer was retained. A random selection was made for any remaining multiple diagnoses such that each patient had only a single tumour included in the analysis. Based on International Statistical Classification of Diseases and Related Health Problems 10th Revision(ICD-10) codes, 25 common sites plus ductal carcinoma in situ were identified with remaining cancers assigned to an ‘other’ category (codes C00-C97 and D05.1, but not C44; online supplemental table 1). Cancer stage was divided into two groups; early stage included cancer stages 0, I and II, and advanced stage included stages III and IV. Brain cancer and leukaemia are not stageable in the system used for staging (tumour, node, metastases), and were therefore excluded from stage analyses (please see online supplemental table 2 for missing stage information).

### Mortality and route to diagnosis

Office for National Statistics death data were used to establish date of death. Based on this date, a binary variable was created that indicated whether a patient had died of any cause within 30 days after their cancer diagnosis. Route to diagnosis data in NCRAS was used to extract pathways leading directly to diagnosis. Examples of these pathways are diagnoses after an urgent suspected cancer referral or emergency presentation.^31^ An emergency presentation includes emergency admission, referral, transfer or attendance to or within secondary care. Two binary variables were created, one indicating whether patients were diagnosed via an emergency presentation (vs any other route), and the other indicating whether patients were diagnosed via urgent suspected cancer referral.

### Data analysis

Logistic regression models were used to investigate whether patients with a learning disability were more likely to: be diagnosed at advanced stage (early (0, I and II) vs advanced stage (III and IV)), be diagnosed via an emergency route (emergency presentation vs other routes), be diagnosed via an urgent suspected cancer referral (urgent referral vs other routes) or die within 30 days of diagnosis. Initial main effects-only models included patient age, sex, cancer site, an indicator variable for learning disability, deprivation quintile, morbidity burden, smoking history and year of diagnosis. Clustering of observations at general practice-level was accounted for by an SE adjustment. In order to investigate cancer site specific differences, two-way interaction terms between cancer site and patient characteristics and cancer site and the presence of a learning disability were investigated individually with all interactions retained in the final model. A joint Wald test was used to test for significance. For most variables included in the models, completeness of records was assumed, with the absence of a record indicating the absence of a diagnosis. Missing data on deprivation (0.03%) and cancer stage (20.5%) was considered missing at random. Analyses with missing outcome data are unbiased under the missing at random assumption. Complete case analysis was therefore conducted for every model. Complete case analysis was considered most appropriate for this study, as previous research comparing a complete case approach with multiple imputation using auxiliary variables, and an approach where those with missing stage are assumed to be late stage suggested that multiple imputation of missing stage does not materially impact findings,^32^ and that an aggregation of late stage with missing stage may introduce greater bias than a complete case analysis.^33^ Analyses were conducted using Stata V.17.^34^

### Role of the funding source

This study is funded by the NIHR Programme Grants for Applied Research SPOCC programme: supporting clinical decision-making in patients with symptoms of cancer and pre-existing conditions (NIHR201070). The funder had no role in study design, data collection, analysis, interpretations or writing of the report.

## Results

We included 277 050 patients in the overall study (table 1), of which 211 423 were included in analyses with cancer stage as outcome (online supplemental table 3). Learning disability was recorded for 796 (0.3%) patients. Patients with a learning disability were generally younger than patients without a learning disability, with an average age of 63 (SD: 11.3; range: 40.1–94.2) compared with 70.2 years (SD: 12.4; range: 40–107.8). The most common cancer sites for patients with a learning disability were colon (n=94; 11.8%) and breast (n=77; 9.7%), while for patients without a learning disability, the most common cancers were prostate (n=44 830; 16.2%) and lung (n=36 687; 13.3%) (table 1).

**Table 1:** Patient characteristics for patients with and without a learning disability

	No learning disability (n=276 254)	Learning disability (n=796)	Total (n=277 050)
	N (%)	N (%)	N (%)
Age			
40–44 years	7117	47	7164
	(2.6)	(5.9)	(2.6)
45–49 years	12 686	67	12 753
	(4.6)	(8.4)	(4.6)
50–54 years	17 048	94	17 142
	(6.2)	(11.8)	(6.2)
55–59 years	22 999	119	23 118
	(8.3)	(15.0)	(8.3)
60–64 years	29 783	131	29 914
	(10.8)	(16.5)	(10.8)
65–69 years	40 268	111	40 379
	(14.6)	(13.9)	(14.6)
70–74 years	41 974	102	42 076
	(15.2)	(12.8)	(15.2)
75–79 years	39 403	69	39 472
	(14.3)	(8.7)	(14.3)
80–84 years	32 610	33	32 643
	(11.8)	(4.2)	(11.8)
85–89 years	21 040	20	21 060
	(7.6)	(2.5)	(7.6)
90 years and older	11 326	3	11 329
	(4.1%	(0.4)	(4.1)
Sex (female)	128 106	360	128 466
	(46.4)	(45.2)	(46.4)
IMD			
1 (least deprived	63 928	93	64 021
	(23.2)	(11.7)	(23.1)
2	59 384	128	59 512
	(21.5)	(16.1)	(21.5)
3	54 112	188	54 300
	(19.6)	(23.6)	(19.6)
4	50 871	174	51 045
	(18.4)	(21.9)	(18.4)
5 (most deprived)	47 878	213	48 091
	(17.3)	(26.8)	(17.4)
Ethnicity			
White	256 125	755	256 880
	(92.7)	(94.9)	(92.7)
Asian	7492	14	7506
	(2.7)	(1.8)	(2.7)
Black	6605	14	6619
	(2.4)	(1.8)	(2.4)
Other	1564	0	1564
	(0.6)	(0.0)	(0.6)
Mixed	1408	7	1415
	(0.5)	(0.9)	(0.5)
Unknown	3060	6	3066
	(1.1)	(0.8)	(1.1)
Morbidity burden			
No morbidity burden	43 951	66	44 017
	(15.9)	(8.3)	(15.9)
Low morbidity burden	77 934	124	78 058
	(28.2)	(15.6)	(28.2)
Medium morbidity burden	77 321	299	77 620
	(28.0)	(37.6)	(28.0)
High morbidity burden	77 048	307	77 355
	(27.9)	(38.6)	(27.9)
Cancer type			
Bladder	7748	21	7769
	(2.8)	(2.6)	(2.8)
Brain	3855	15	3870
	(1.4)	(1.9)	(1.4)
Breast	29 753	77	29 830
	(10.8)	(9.7)	(10.8)
Cervix	1266	2	1268
	(0.5)	(0.3)	(0.5)
Colon	21 330	94	21 424
	(7.7)	(11.8)	(7.7)
DCIS	2622	6	2628
	(1.0)	(0.8)	(1.0)
Hodgkin lymphoma	1025	2	1027
	(0.4)	(0.3)	(0.4)
Larynx	1731	2	1733
	(0.6)	(0.3)	(0.6)
Leukaemia	7071	23	7094
	(2.6)	(2.9)	(2.6)
Liver	4919	20	4939
	(1.8)	(2.5)	(1.8)
Lung	36 687	62	36 749
	(13.3)	(7.8)	(13.3)
Melanoma	11 060	22	11 082
	(4.0)	(2.8)	(4.0)
Mesothelioma	2261	4	2265
	(0.8)	(0.5)	(0.8)
Myeloma	4907	10	4917
	(1.8)	(1.3)	(1.8)
Non-Hodgkin lymphoma	10 965	34	10 999
	(4.0)	(4.3)	(4.0)
Oesophagus	7780	36	7816
	(2.8)	(4.5)	(2.8)
Oral	7061	10	7071
	(2.6)	(1.3)	(2.6)
Other	18 544	101	18 645
	(6.7)	(12.7)	(6.7)
Ovary	6111	29	6140
	(2.2)	(3.6)	(2.2)
Pancreas	8566	18	8584
	(3.1)	(2.3)	(3.1)
Prostate	44 830	57	44 887
	(16.2)	(7.2)	(16.2)
Rectum	10 938	48	10 986
	(4.0)	(6.0)	(4.0)
Renal	8669	28	8697
	(3.1)	(3.5)	(3.1)
Stomach	5393	18	5411
	(2.0)	(2.3)	(2.0)
Testis	895	7	902
	(0.3)	(0.9)	(0.3)
Thyroid	2304	3	2307
	(0.8)	(0.4)	(0.8)
Uterus	7963	47	8010
	(2.9)	(5.9)	(2.9)

DCIS, ductal carcinoma in situ; IMD, Index of Multiple Deprivation.

Patients with a learning disability had higher rates of emergency diagnoses (36.4% (n=290) compared with 20.4% (n=56 209)) and fewer diagnoses via an urgent suspected cancer referral than patients without a learning disability (25.6% (n=204) compared with 40.7% (n=112 338)). Patients with a learning disability also had a higher number of advanced stage diagnoses (53.9% (n=282) compared with 50.2% (n=105 763) (online supplemental table 2 for missing stage information) and higher 30-day mortality rates (18.8% (n=150) compared with 8.5% (n=23 353)) than patients without a learning disability (table 2).

**Table 2:** Outcomes and routes to diagnosis for patients with and without a learning disability

	No learning disability (n=276 254)	Learning disability (n=796)	Total (n=277 050)
			N (%)
Outcomes			
Emergency presentation route to diagnosis	56 209	290	56 499
	(20.4%)	(36.4%)	(20.4)
Urgent suspected cancer referral route to diagnosis	112 338	204	112 542
	(40.7%)	(25.6%)	(40.6)
30-day mortality post-diagnosis of cancer	23 353	150	23 503
	(8.5%)	(18.8%)	(8.5)
	N=210 900*	N=523*	N=211 423*
Advanced-stage cancer diagnosis	105 763	282	106 045
	(50.2%)	(53.9%)	(50.2)

* 266 086 patients had a recorded diagnosis of a stageable cancer in NCRAS. Information on stage was provided for 211 423 (79.5%) of these patients. The number of patients for stage is therefore lower than for other outcomes (online supplemental table 2 for information on missing stage).

NCRAS, National Cancer Registration and Analysis Service.

### Routes to diagnosis

After adjusting for age, sex, deprivation, morbidity burden, smoking history, cancer site and year of diagnosis, patients with a learning disability were much more likely to be diagnosed via emergency presentations (adjusted OR (aOR): 2.65, 95% CI 2.26 to 3.11, p<0.001; online supplemental table 4), and much less likely via urgent suspected cancer referrals (aOR: 0.51, 95% CI 0.43 to 0.60, p<0.001; online supplemental table 5) than patients without a learning disability (table 3). The interaction effect model for emergency presentation found evidence (p<0.05) that patients with a learning disability were more likely to be diagnosed after an emergency presentation for the majority of cancer sites (online supplemental figure 2), while an interaction effects model for urgent suspected cancer referral found evidence (p<0.05) that patients with a learning disability who were diagnosed with oral, lung, ovarian, prostate, uterus, other, rectum, colon, testicular and breast cancer were less likely to be diagnosed after an urgent suspected cancer referral than patients without a learning disability (online supplemental figure 3).

**Table 3:** Associations between emergency presentation route to diagnosis, urgent referral route to diagnosis, cancer stage at diagnosis, 30-day mortality and learning disability

	Learning disability (ref.: no learning disability)*			
	OR	Lower 95% CI	Upper 95% CI	P value
Outcomes				
Emergency presentation route to diagnosis	2.65	2.26	3.11	<0.001
Urgent suspected cancer referral route to diagnosis	0.51	0.43	0.60	<0.001
Advanced-stage cancer diagnosis	1.37	1.12	1.67	0.002
30-day mortality post-diagnosis of cancer	3.77	3.10	4.59	<0.001

* Separate regression models were run per outcome. Analyses were adjusted for patient age, sex, cancer site, an indicator variable for learning disability, deprivation quintile, morbidity burden, smoking history and year of diagnosis.

### Cancer stage and mortality

After adjusting for age, sex, deprivation, morbidity burden, smoking history, cancer site and year of diagnosis, patients with a learning disability were more likely to be diagnosed with advanced-stage cancer (aOR: 1.37, 95% CI 1.12 to 1.67, p=0.002; online supplemental table 6) and to die within 30 days after their diagnosis (aOR: 3.77, 95% CI 3.10 to 4.59, p<0.001; online supplemental table 7) than patients without a learning disability (table 3). Interaction effects models for cancer stage and mortality found evidence (p<0.05) that effects of learning disability vary by cancer site. For stage, there was evidence that patients with a learning disability were far more likely to be diagnosed at advanced stage for breast (aOR: 3.36, 95% CI 1.99 to 5.65, p<0.001) and ‘other’ cancers (aOR: 3.87, 95% CI 1.34 to 11.17, p=0.012) (online supplemental figure 4). For mortality, patients with a learning disability diagnosed with all cancers apart from uterus, stomach, pancreatic and rectal cancer and non-Hodgkin lymphoma, were more likely to die within 30 days of their cancer diagnosis than patients without a learning disability (online supplemental figure 5). Large uncertainty due to limited numbers meant that many substantial point estimates for individual cancer sites did not reach statistical significance and it should be noted that for some sites point estimates were consistent with patients with a learning disability being diagnosed at earlier stages than patients without (oral, uterus, bladder, pancreas, mesothelioma, renal, stomach and melanoma) (online supplemental figure 4). For 30-day mortality, all point estimates favoured those without a learning disability.

## Discussion

### Statement of principal findings

The present study investigated whether patients with a learning disability diagnosed with any cancer experience inequalities in diagnostic processes and outcomes. We found that patients with a learning disability experience significant disadvantages; having over two times the odds of being diagnosed via an emergency diagnosis and nearly half the odds via the preferred route of an urgent suspected cancer referral, compared with patients without a learning disability. This study also found that patients with a learning disability are 37% more likely to be diagnosed at an advanced stage—over three times as likely for breast cancer—and have close to four times the odds of dying within 30 days after their cancer diagnosis.

### Comparison with existing literature

Previous research suggests that patients with a learning disability may experience difficulties in communicating their symptoms, and diagnostic overshadowing—where symptoms of a condition are erroneously attributed to their learning disability.^15 16 35^ This could result in the cancer not being recognised by the carer, or in primary care, which potentially contributes to patients not being referred for cancer investigations and an increase in emergency diagnoses once the symptoms become urgent. To better understand whether possible cancer symptoms are not acted on in primary care, research is needed to investigate presenting patterns and resulting GP actions for patients with a learning disability. An alternative explanation for our findings is that the patient, carer and/or healthcare professional decide to not pursue cancer investigations,^15^ and the diagnosis is made once the patient presents as an emergency. Even when adjusted for cancer stage, emergency diagnosis is commonly linked to worse patient outcomes.^31 36^ It is therefore important for future research to provide more insight into how patients with a learning disability come to be diagnosed as an emergency, and for carers and healthcare professionals to support cancer diagnosis through other routes.

The present study also found that patients with a learning disability are more likely to be diagnosed at an advanced stage, particularly if they are diagnosed with breast cancer, for which their odds are over threefold that of those without a learning disability. Existing evidence surrounding diagnostic outcomes is limited, but our findings are in line with previous research suggesting that patients with a learning disability are generally more likely to be diagnosed at an advanced stage,^37 38^ and one small study suggesting that patients with breast cancer with a learning disability were more likely to be diagnosed at an advanced stage than those without.^39^ The finding regarding breast cancer is especially notable, as, although a greater likelihood of being diagnosed with advanced cancer could in part be explained by a lower uptake of screening,^40^ a previous study found that for most patients breast cancer is generally quickly identified in primary care.^41^ Future research should investigate whether associations of breast cancer symptoms with incident cancer are different for patients with and without a learning disability and whether health care professionals act differently when patients with a learning disability present with such symptoms. In addition, improved support for patients with a learning disability to attend screening, and increased alertness to patients with a learning disability who present with possible breast cancer symptoms in primary care may help diagnose breast cancer earlier.

On top of an increased likelihood of being diagnosed with advanced-stage cancer, our results suggest that patients with a learning disability also experienced increased mortality shortly after being diagnosed with cancer. Although earlier research indicated that patients with a learning disability had greater cancer mortality rates than patients without a learning disability,^38 42 43^ no studies have previously investigated short-term mortality. We do not yet know to what extent increased mortality is caused by; (1) patients with a learning disability being diagnosed at a point where cancer is an immediate threat to life; (2) cancer being diagnosed during emergency presentations for other life threatening conditions; (3) the presence of a learning disability affecting short-term care; (4) or a lower life expectancy for patients with a learning disability.^44^ Our findings of suboptimal pathways to diagnosis and poor outcomes combined with previous findings regarding low cancer awareness, and difficulties navigating the diagnostic process,^15 17^ however, indicate a need for significant improvements in cancer awareness and diagnostic processes for patients with a learning disability.

### Strengths and weaknesses of the study

This is, to our knowledge, the first English retrospective cohort study using healthcare records to investigate diagnostic pathways and outcomes for patients with a learning disability diagnosed with any cancer. CPRD linked data are generally considered to be a representative sample of the consulting population in England, thus allowing inferences about our study’s results for the English population.^18 22^ Some limitations, however, may have affected our findings. First, the work described here is a small part of a work package embedded in a Programme Grant. The work package aimed to explore the diagnosis of cancer in patients with pre-existing health conditions and studied 10 outcomes and 121 health conditions. The results for learning disability are discussed here, as this group experienced the worst outcomes of all patient groups, and the findings are likely to be of particular interest to researchers and policymakers. However, these findings should be considered in the light of the multiple analyses undertaken.

Only 0.3% of our study population had a record of a learning disability, which is somewhat lower than that seen in previous evidence suggesting that health records reflect up to a 0.5% prevalence of learning disabilities in the English population.^15 45 46^ This likely reflects the age of patients with cancer and lower life expectancy of those with learning disabilities. Even so, estimates from health records are considerably lower than previous suggestions that approximately 2.5% of the English population has a learning disability.^15^ It is possible that recordings of learning disabilities in health records were missed as they were recorded in ‘free/uncoded text’, which is not accessible to researchers. There is, however, generally a high degree of overlap between free-text and coded records.^47^ English GP practices are also financially incentivised to keep a register of patients with a learning disability, so coded recording is likely to be high. A more likely cause for the low prevalence is that the code list for registering patients with a learning disability (which was used in this study) only includes disorders where the estimated probability of having a learning disability is 80% or higher.^29^ Thus, we may have captured fewer cases with milder learning disability. We were not able to take into account the severity of the learning disability, or the presence of other complicating factors such as coexisting autism or severe mental illness, which are more common in people with a learning disability.^48 49^ Our findings nevertheless reflect the cancer diagnostic outcomes of patients included on English GP practices’ registers of people with a learning disability.

Though we captured data on all cases in the cancer registry data with linked primary care data over a 7-year period, small sample sizes for individual cancer sites and in particular for less common sites, meant that many estimates did not reach statistical significance. The absence of statistically significant associations should not be treated as evidence that such an association does not exist.

## Conclusion

Patients with a learning disability experience marked inequalities in cancer diagnostic pathways and outcomes. They are more likely to experience unfavourable routes to diagnosis, increased short-term mortality and are more likely to be diagnosed at an advanced stage than patients without a learning disability. Previous evidence suggested that patients may have low symptom awareness and experience difficulties accessing and navigating care, which may have contributed to the inequalities highlighted by this study. Greater efforts need to be made to improve cancer awareness and support patients with a learning disability to access and navigate the healthcare system to improve cancer diagnostic outcomes.

## Supplementary material

10.1136/bmjonc-2026-001104 online supplemental appendix 1

## Data Availability

Data may be obtained from a third party and are not publicly available.
