# Supplementary material for: Inequalities in cancer diagnostic outcomes for patients with a learning disability: a retrospective cohort study in England
Source: BMJ Oncol. 2026 May 18;5(1):e001104. doi: 10.1136/bmjonc-2026-001104 (PMC13202048; doi:10.1136/bmjonc-2026-001104)
Supplement: online supplemental appendix 1 [file bmjonc-5-1-s001.docx]

## Appendix A

### Tables

**Table 1. ICD-10 codes for each cancer site included in the study**

| **Cancer site** | **ICD-10 code** |
| --- | --- |
| Lip, oral cavity and pharynx | C00, C01, C02, C03, C04, C05, C06, C07, C08, C09, C10, C11, C12, C13, C14 |
| Oesophagus | C15 |
| Liver | C22 |
| Stomach | C16 |
| Colon | C18 |
| Rectum | C19, C20 |
| Pancreas | C25 |
| Larynx | C32 |
| Lung | C33, C34 |
| Melanoma | C43 |
| Mesothelioma | C45 |
| Breast | C50 |
| Cervix | C53 |
| Uterus | C54, C55 |
| Ovary | C56, C57 |
| Prostate | C61 |
| Testis | C62 |
| Kidney | C64 |
| Bladder | C67 |
| Brain | C71 |
| Thyroid | C73 |
| Hodgkin lymphoma | C81 |
| Non-Hodgkin lymphoma | C82, C83, C84, C85 |
| Myeloma | C90 |
| Leukaemia | C91, C92, C93, C94, C95 |
| DCIS | D05.1 |

**Table 2.** **Missing cancer stage for stageable cancers for patients with and without a learning disability**

|  | **Stageable cancers** | **Missing cancer stage for stageable cancers** |
| --- | --- | --- |
|  | N (%) | N (%)* |
| No learning disability | 265328  (99.7%) | 54428  (20.5%) |
| Learning disability | 758  (0.3%) | 235  (31.0%) |
| Total | 266086  (100%) | 54663  (20.5%) |

*Percentages presented in this column are row percentages and indicate the percentage of missing stage records for stageable cancers per group.

**Table 3. Patient characteristics and outcomes for patients with recorded cancer stage**

|  | No learning disability (n=210,900) | Learning disability (n=523) | Total (n=211,423) |
| --- | --- | --- | --- |
|  | N (%) | N (%) | N (%) |
| *Age* |  |  |  |
| 40 to 44 years | 5629 | 35 | 5664 |
|  | (2.7%) | (6.7%) | (2.7%) |
| 45 to 49 years | 10138 | 47 | 10185 |
|  | (4.8%) | (9.0%) | (4.8%) |
| 50 to 54 years | 13677 | 67 | 13744 |
|  | (6.5%) | (12.8%) | (6.5%) |
| 55 to 59 years | 18490 | 85 | 18575 |
|  | (8.8%) | (16.3%) | (8.8%) |
| 60 to 64 years | 24010 | 84 | 24094 |
|  | (11.4%) | (16.1%) | (11.4%) |
| 65 to 69 years | 32528 | 72 | 32600 |
|  | (15.4%) | (13.8%) | (15.4%) |
| 70 to 74 years | 33685 | 70 | 33755 |
|  | (16.0%) | (13.4%) | (16.0%) |
| 75 to 79 years | 30504 | 35 | 30539 |
|  | (14.5%) | (6.7%) | (14.4%) |
| 80 to 84 years | 23207 | 16 | 23223 |
|  | (11.0%) | (3.1%) | (11.0%) |
| 85 to 89 years | 13292 | 11 | 13303 |
|  | (6.3%) | (2.1%) | (6.3%) |
| 90 years and older | 5740 | 1 | 5741 |
|  | (2.7%) | (0.2%) | (2.7%) |
|  |  |  |  |
| Sex (Female) | 97739 | 224 | 97963 |
|  | (46.3%) | (42.8%) | (46.3%) |
|  |  |  |  |
| *IMD* | 48293 | 71 | 48364 |
| 1 (least deprived | (22.9%) | (13.6%) | (22.9%) |
|  | 45256 | 81 | 45337 |
| 2 | (21.5%) | (15.5%) | (21.5%) |
|  | 41312 | 119 | 41431 |
| 3 | (19.6%) | (22.8%) | (19.6%) |
|  | 39008 | 115 | 39123 |
| 4 | (18.5%) | (22.0%) | (18.5%) |
|  | 36970 | 137 | 37107 |
| 5 (most deprived) | (17.5%) | (26.2%) | (17.6%) |
|  |  |  |  |
|  |  |  |  |
| *Ethnicity* |  |  |  |
| White | 195831 | 489 | 196320 |
|  | (92.9%) | (93.5%) | (92.9%) |
| Asian | 5597 | 13 | 5610 |
|  | (2.7%) | (2.5%) | (2.7%) |
| Black | 5100 | 10 | 5110 |
|  | (2.4%) | (1.9%) | (2.4%) |
| Other | 1194 | 0 | 1194 |
|  | (0.6%) | (0.0%) | (0.6%) |
| Mixed | 1115 | 7 | 1122 |
|  | (0.5%) | (1.3%) | (0.5%) |
| Unknown | 2063 | 4 | 2067 |
|  | (1.0%) | (0.8%) | (1.0%) |
|  |  |  |  |
| *Morbidity burden* |  |  |  |
| No morbidity burden | 34716 | 47 | 34763 |
|  | (16.5%) | (9.0%) | (16.4%) |
| Low morbidity burden | 61817 | 87 | 61904 |
|  | (29.3%) | (16.6%) | (29.3%) |
| Medium morbidity burden | 59238 | 194 | 59432 |
|  | (28.1%) | (37.1%) | (28.1%) |
| High morbidity burden | 55129 | 195 | 55324 |
|  | (26.1%) | (37.3%) | (26.2%) |
|  |  |  |  |
| *Cancer type* |  |  |  |
| Bladder | 6090 | 12 | 6102 |
|  | (2.9%) | (2.3%) | (2.9%) |
| Breast | 26271 | 56 | 26327 |
|  | (12.5%) | (10.7%) | (12.5%) |
| Cervix | 922 | 1 | 923 |
|  | (0.4%) | (0.2%) | (0.4%) |
| Colon | 18714 | 84 | 18798 |
|  | (8.9%) | (16.1%) | (8.9%) |
| DCIS | 2621 | 6 | 2627 |
|  | (1.2%) | (1.2%) | (1.2%) |
| HL | 850 | 2 | 852 |
|  | (0.4%) | (0.4%) | (0.4%) |
| Larynx | 1409 | 1 | 1410 |
|  | (0.7%) | (0.2%) | (0.7%) |
| Liver | 2114 | 8 | 2122 |
|  | (1.0%) | (1.5%) | (1.0%) |
| Lung | 33324 | 53 | 33377 |
|  | (15.8%) | (10.1%) | (15.8%) |
| Melanoma | 9943 | 21 | 9964 |
|  | (4.7%) | (4.0%) | (4.7%) |
| Mesothelioma | 1010 | 2 | 1012 |
|  | (0.5%) | (0.4%) | (0.5%) |
| Myeloma | 1124 | 1 | 1125 |
|  | (0.5%) | (0.2%) | (0.5%) |
| NHL | 8325 | 26 | 8351 |
|  | (4.0%) | (5.0%) | (4.0%) |
| Oesophagus | 6166 | 26 | 6192 |
|  | (2.9%) | (5.0%) | (2.9%) |
| Oral | 5935 | 8 | 5943 |
|  | (2.8%) | (1.5%) | (2.8%) |
| Other | 6093 | 25 | 6118 |
|  | (2.9%) | (4.8%) | (2.9%) |
| Ovary | 4976 | 15 | 4991 |
|  | (2.4%) | (2.9%) | (2.4%) |
| Pancreas | 6071 | 14 | 6085 |
|  | (2.9%) | (2.7%) | (2.9%) |
| Prostate | 38997 | 48 | 39045 |
|  | (18.5%) | (9.2%) | (18.5%) |
| Rectum | 9784 | 37 | 9821 |
|  | (4.6%) | (7.1%) | (4.7%) |
| Renal | 6990 | 21 | 7011 |
|  | (3.3%) | (4.0%) | (3.3%) |
| Stomach | 4031 | 8 | 4039 |
|  | (1.9%) | (1.5%) | (1.9%) |
| Testis | 670 | 6 | 676 |
|  | (0.3%) | (1.2%) | (0.3%) |
| Thyroid | 1462 | 3 | 1465 |
|  | (0.7%) | (0.6%) | (0.7%) |
| Uterus | 7008 | 39 | 7047 |
|  | (3.3%) | (7.5%) | (3.3%) |
|  |  |  |  |
| Emergency presentation route to diagnosis | 34515 | 157 | 34672 |
|  | (16.4%) | (30.0%) | (16.4%) |
| Urgent suspected cancer referral route to diagnosis | 98889 | 170 | 99059 |
|  | (46.9%) | (32.5%) | (46.9%) |
| 30-day mortality | 10692 | 55 | 10747 |
|  | (5.1%) | (10.5%) | (5.1%) |

### **Table 4. Main effect only model associations between emergency presentation route to diagnosis, patient characteristics, cancer type and learning disability**

|  | **Emergency presentation route to diagnosis** | | | | |  | | | |
| --- | --- | --- | --- | --- | --- | --- | --- | --- | --- |
|  | Un-adjusted odds ratio | Lower 95% confidence interval | Upper 95% confidence interval | P | Adjusted odds ratio | | Lower 95% confidence interval | Upper 95% confidence interval | P |
| *Cancer type* |  |  |  | P<0.001 |  | |  |  | P<0.001 |
| Bladder | 0.46 | 0.43 | 0.49 |  | 0.42 | | 0.39 | 0.45 |  |
| Brain | 2.63 | 2.44 | 2.83 |  | 3.49 | | 3.23 | 3.77 |  |
| Breast | 0.14 | 0.13 | 0.15 |  | 0.14 | | 0.14 | 0.15 |  |
| Cervix | 0.45 | 0.38 | 0.52 |  | 0.55 | | 0.47 | 0.65 |  |
| Colon | 1.00 | 1.00 | 1.00 |  | 1.00 | | 1.00 | 1.00 |  |
| DCIS | 0.06 | 0.05 | 0.08 |  | 0.08 | | 0.06 | 0.10 |  |
| HL | 0.42 | 0.35 | 0.50 |  | 0.55 | | 0.46 | 0.66 |  |
| Larynx | 0.25 | 0.21 | 0.29 |  | 0.28 | | 0.24 | 0.34 |  |
| Leukaemia | 1.00 | 0.94 | 1.07 |  | 1.08 | | 1.01 | 1.16 |  |
| Liver | 1.66 | 1.56 | 1.76 |  | 1.71 | | 1.60 | 1.83 |  |
| Lung | 1.20 | 1.15 | 1.24 |  | 1.18 | | 1.13 | 1.23 |  |
| Melanoma | 0.04 | 0.04 | 0.05 |  | 0.05 | | 0.05 | 0.06 |  |
| Mesothelioma | 1.14 | 1.04 | 1.25 |  | 1.17 | | 1.07 | 1.29 |  |
| Myeloma | 1.09 | 1.02 | 1.17 |  | 1.19 | | 1.11 | 1.28 |  |
| NHL | 0.76 | 0.72 | 0.80 |  | 0.86 | | 0.82 | 0.91 |  |
| Oesophagus | 0.53 | 0.49 | 0.56 |  | 0.54 | | 0.50 | 0.57 |  |
| Oral | 0.16 | 0.15 | 0.18 |  | 0.20 | | 0.18 | 0.22 |  |
| Other | 1.34 | 1.29 | 1.40 |  | 1.34 | | 1.28 | 1.40 |  |
| Ovary | 0.90 | 0.85 | 0.96 |  | 1.08 | | 1.01 | 1.15 |  |
| Pancreas | 1.71 | 1.62 | 1.80 |  | 1.78 | | 1.69 | 1.88 |  |
| Prostate | 0.17 | 0.16 | 0.17 |  | 0.19 | | 0.18 | 0.20 |  |
| Rectum | 0.32 | 0.30 | 0.34 |  | 0.34 | | 0.32 | 0.37 |  |
| Renal | 0.61 | 0.57 | 0.64 |  | 0.69 | | 0.65 | 0.74 |  |
| Stomach | 0.99 | 0.93 | 1.06 |  | 0.96 | | 0.89 | 1.02 |  |
| Testis | 0.16 | 0.13 | 0.21 |  | 0.26 | | 0.20 | 0.34 |  |
| Thyroid | 0.17 | 0.15 | 0.20 |  | 0.24 | | 0.20 | 0.28 |  |
| Uterus | 0.18 | 0.17 | 0.20 |  | 0.22 | | 0.20 | 0.24 |  |
|  |  |  |  |  |  | |  |  |  |
| Learning disability (yes) | 2.24 | 1.94 | 2.60 | P<0.001 | 2.65 | | 2.26 | 3.11 | P<0.001 |
|  |  |  |  |  |  | |  |  |  |
| *Morbidity burden* |  |  |  | P<0.001 |  | |  |  | P<0.001 |
| No morbidity burden | 1.00 | 1.00 | 1.00 |  | 1.00 | | 1.00 | 1.00 |  |
| Low morbidity burden | 1.00 | 0.96 | 1.03 |  | 0.87 | | 0.84 | 0.91 |  |
| Medium morbidity burden | 1.41 | 1.37 | 1.46 |  | 0.95 | | 0.92 | 0.99 |  |
| High morbidity burden | 2.26 | 2.19 | 2.34 |  | 1.18 | | 1.14 | 1.23 |  |
|  |  |  |  |  |  | |  |  |  |
| Sex (Female) | 1.11 | 1.08 | 1.13 | P<0.001 | 1.02 | | 1.00 | 1.05 | 0.0397 |
|  |  |  |  |  |  | |  |  |  |
| *Age* |  |  |  | P<0.001 |  | |  |  | P<0.001 |
| 40 to 44 years | 0.63 | 0.58 | 0.68 |  | 0.87 | | 0.80 | 0.94 |  |
| 45 to 49 years | 0.69 | 0.65 | 0.73 |  | 0.92 | | 0.86 | 0.98 |  |
| 50 to 54 years | 0.78 | 0.74 | 0.83 |  | 0.92 | | 0.87 | 0.97 |  |
| 55 to 59 years | 0.88 | 0.84 | 0.92 |  | 0.94 | | 0.90 | 0.99 |  |
| 60 to 64 years | 0.93 | 0.89 | 0.97 |  | 0.95 | | 0.91 | 1.00 |  |
| 65 to 69 years | 1.00 | 1.00 | 1.00 |  | 1.00 | | 1.00 | 1.00 |  |
| 70 to 74 years | 1.19 | 1.15 | 1.24 |  | 1.15 | | 1.10 | 1.19 |  |
| 75 to 79 years | 1.42 | 1.37 | 1.47 |  | 1.32 | | 1.27 | 1.37 |  |
| 80 to 84 years | 2.00 | 1.93 | 2.07 |  | 1.78 | | 1.71 | 1.84 |  |
| 85 to 89 years | 2.97 | 2.85 | 3.09 |  | 2.67 | | 2.56 | 2.79 |  |
| 90 years and older | 4.47 | 4.26 | 4.69 |  | 4.44 | | 4.21 | 4.68 |  |
|  |  |  |  |  |  | |  |  |  |
| *IMD* |  |  |  | P<0.001 |  | |  |  | P<0.001 |
| 1 (least deprived | 1.00 | 1.00 | 1.00 |  | 1.00 | | 1.00 | 1.00 |  |
| 2 | 1.13 | 1.10 | 1.17 |  | 1.09 | | 1.05 | 1.12 |  |
| 3 | 1.24 | 1.20 | 1.29 |  | 1.16 | | 1.12 | 1.20 |  |
| 4 | 1.42 | 1.37 | 1.47 |  | 1.28 | | 1.24 | 1.33 |  |
| 5 (most deprived) | 1.62 | 1.57 | 1.68 |  | 1.42 | | 1.37 | 1.47 |  |
|  |  |  |  |  |  | |  |  |  |
| Smoking history | 1.29 | 1.26 | 1.31 |  | 1.02 | | 1.00 | 1.05 | 0.0674 |
|  |  |  |  |  |  | |  |  |  |
| *Year of diagnosis* |  |  |  | P<0.001 |  | |  |  | P<0.001 |
| 2012 | 1.00 | 1.00 | 1.00 |  | 1.00 | | 1.00 | 1.00 |  |
| 2013 | 0.96 | 0.93 | 0.99 |  | 0.95 | | 0.92 | 0.99 |  |
| 2014 | 0.96 | 0.93 | 1.00 |  | 0.97 | | 0.93 | 1.00 |  |
| 2015 | 0.90 | 0.87 | 0.94 |  | 0.91 | | 0.87 | 0.94 |  |
| 2016 | 0.87 | 0.84 | 0.90 |  | 0.87 | | 0.84 | 0.91 |  |
| 2017 | 0.83 | 0.80 | 0.86 |  | 0.83 | | 0.80 | 0.86 |  |
| 2018 | 0.81 | 0.78 | 0.83 |  | 0.84 | | 0.81 | 0.87 |  |

**Table 5. Main effect only model associations between urgent (two-week-wait) referral route to diagnosis, patient characteristics, cancer type and learning disability**

|  | **Urgent suspected cancer referral route to diagnosis** | | | | |  | | | |
| --- | --- | --- | --- | --- | --- | --- | --- | --- | --- |
|  | Un-adjusted odds ratio | Lower 95% confidence interval | Upper 95% confidence interval | P | Adjusted odds ratio | | Lower 95% confidence interval | Upper 95% confidence interval | P |
| *Cancer type* |  |  |  | P<0.001 |  | |  |  | P<0.001 |
| Bladder | 2.14 | 2.02 | 2.26 |  | 2.16 | | 2.04 | 2.28 |  |
| Brain | 0.04 | 0.03 | 0.05 |  | 0.03 | | 0.03 | 0.04 |  |
| Breast | 7.12 | 6.80 | 7.45 |  | 7.55 | | 7.20 | 7.92 |  |
| Cervix | 1.38 | 1.23 | 1.56 |  | 1.38 | | 1.22 | 1.56 |  |
| Colon | 1.00 | 1.00 | 1.00 |  | 1.00 | | 1.00 | 1.00 |  |
| DCIS | 1.74 | 1.60 | 1.90 |  | 1.74 | | 1.60 | 1.90 |  |
| HL | 1.40 | 1.23 | 1.59 |  | 1.36 | | 1.20 | 1.55 |  |
| Larynx | 2.45 | 2.20 | 2.72 |  | 2.32 | | 2.08 | 2.58 |  |
| Leukaemia | 0.39 | 0.36 | 0.42 |  | 0.38 | | 0.35 | 0.41 |  |
| Liver | 0.39 | 0.36 | 0.42 |  | 0.39 | | 0.36 | 0.42 |  |
| Lung | 0.89 | 0.85 | 0.93 |  | 0.88 | | 0.84 | 0.92 |  |
| Melanoma | 4.21 | 3.99 | 4.44 |  | 4.14 | | 3.92 | 4.37 |  |
| Mesothelioma | 0.73 | 0.66 | 0.81 |  | 0.70 | | 0.63 | 0.78 |  |
| Myeloma | 0.62 | 0.58 | 0.67 |  | 0.61 | | 0.57 | 0.66 |  |
| NHL | 0.88 | 0.84 | 0.93 |  | 0.86 | | 0.82 | 0.90 |  |
| Oesophagus | 1.87 | 1.77 | 1.98 |  | 1.85 | | 1.75 | 1.96 |  |
| Oral | 2.38 | 2.24 | 2.52 |  | 2.29 | | 2.16 | 2.42 |  |
| Other | 0.54 | 0.51 | 0.56 |  | 0.55 | | 0.52 | 0.57 |  |
| Ovary | 1.14 | 1.08 | 1.22 |  | 1.13 | | 1.07 | 1.21 |  |
| Pancreas | 0.55 | 0.51 | 0.58 |  | 0.54 | | 0.51 | 0.58 |  |
| Prostate | 2.09 | 2.01 | 2.18 |  | 1.94 | | 1.86 | 2.02 |  |
| Rectum | 1.94 | 1.85 | 2.04 |  | 1.88 | | 1.79 | 1.98 |  |
| Renal | 0.94 | 0.89 | 0.99 |  | 0.92 | | 0.87 | 0.97 |  |
| Stomach | 1.02 | 0.95 | 1.08 |  | 1.02 | | 0.96 | 1.09 |  |
| Testis | 3.59 | 3.11 | 4.14 |  | 3.56 | | 3.08 | 4.12 |  |
| Thyroid | 0.72 | 0.66 | 0.80 |  | 0.72 | | 0.65 | 0.79 |  |
| Uterus | 3.03 | 2.86 | 3.21 |  | 2.98 | | 2.81 | 3.16 |  |
|  |  |  |  |  |  | |  |  |  |
| Learning disability (yes) | 0.50 | 0.43 | 0.59 | P<0.001 | 0.51 | | 0.43 | 0.60 | P<0.001 |
|  |  |  |  |  |  | |  |  |  |
| *Morbidity burden* |  |  |  | P<0.001 |  | |  |  | P<0.001 |
| No morbidity burden | 1.00 | 1.00 | 1.00 |  | 1.00 | | 1.00 | 1.00 |  |
| Low morbidity burden | 0.93 | 0.91 | 0.95 |  | 0.95 | | 0.93 | 0.97 |  |
| Medium morbidity burden | 0.81 | 0.79 | 0.83 |  | 0.91 | | 0.89 | 0.94 |  |
| High morbidity burden | 0.61 | 0.59 | 0.62 |  | 0.75 | | 0.73 | 0.77 |  |
|  |  |  |  |  |  | |  |  |  |
| Sex (Female) | 1.24 | 1.22 | 1.27 | P<0.001 | 0.97 | | 0.95 | 0.99 | 0.0144 |
|  |  |  |  |  |  | |  |  |  |
| *Age* |  |  |  | P<0.001 |  | |  |  | P<0.001 |
| 40 to 44 years | 1.30 | 1.24 | 1.37 |  | 0.79 | | 0.74 | 0.84 |  |
| 45 to 49 years | 1.35 | 1.29 | 1.41 |  | 0.83 | | 0.79 | 0.87 |  |
| 50 to 54 years | 1.11 | 1.07 | 1.15 |  | 0.84 | | 0.80 | 0.87 |  |
| 55 to 59 years | 1.03 | 1.00 | 1.07 |  | 0.90 | | 0.87 | 0.94 |  |
| 60 to 64 years | 1.02 | 0.99 | 1.05 |  | 0.96 | | 0.93 | 0.99 |  |
| 65 to 69 years | 1.00 | 1.00 | 1.00 |  | 1.00 | | 1.00 | 1.00 |  |
| 70 to 74 years | 0.97 | 0.95 | 1.00 |  | 1.01 | | 0.98 | 1.04 |  |
| 75 to 79 years | 0.98 | 0.95 | 1.01 |  | 1.05 | | 1.01 | 1.08 |  |
| 80 to 84 years | 0.83 | 0.80 | 0.85 |  | 0.91 | | 0.88 | 0.94 |  |
| 85 to 89 years | 0.68 | 0.65 | 0.70 |  | 0.74 | | 0.71 | 0.77 |  |
| 90 years and older | 0.50 | 0.47 | 0.52 |  | 0.49 | | 0.46 | 0.51 |  |
|  |  |  |  |  |  | |  |  |  |
| *IMD* |  |  |  | P<0.001 |  | |  |  | P<0.001 |
| 1 (least deprived | 1.00 | 1.00 | 1.00 |  | 1.00 | | 1.00 | 1.00 |  |
| 2 | 1.02 | 0.99 | 1.05 |  | 1.07 | | 1.04 | 1.10 |  |
| 3 | 1.02 | 0.99 | 1.05 |  | 1.11 | | 1.07 | 1.15 |  |
| 4 | 0.95 | 0.92 | 0.98 |  | 1.07 | | 1.03 | 1.11 |  |
| 5 (most deprived) | 0.89 | 0.86 | 0.92 |  | 1.06 | | 1.02 | 1.10 |  |
|  |  |  |  |  |  | |  |  |  |
| Smoking history | 0.89 | 0.87 | 0.90 | P<0.001 | 1.08 | | 1.06 | 1.11 | P<0.001 |
|  |  |  |  |  |  | |  |  |  |
| *Year of diagnosis* |  |  |  | P<0.001 |  | |  |  | P<0.001 |
| 2012 | 1.00 | 1.00 | 1.00 |  | 1.00 | | 1.00 | 1.00 |  |
| 2013 | 1.01 | 0.98 | 1.04 |  | 1.02 | | 0.99 | 1.05 |  |
| 2014 | 1.06 | 1.03 | 1.09 |  | 1.07 | | 1.03 | 1.11 |  |
| 2015 | 1.13 | 1.09 | 1.16 |  | 1.15 | | 1.11 | 1.19 |  |
| 2016 | 1.19 | 1.15 | 1.23 |  | 1.23 | | 1.19 | 1.27 |  |
| 2017 | 1.23 | 1.19 | 1.27 |  | 1.28 | | 1.24 | 1.33 |  |
| 2018 | 1.30 | 1.26 | 1.35 |  | 1.34 | | 1.30 | 1.39 |  |

### **Table 6. Main effect only model associations between cancer stage, patient characteristics, cancer type and learning disability**

|  | **Advanced-stage cancer diagnosis** | | | | |  | | | |
| --- | --- | --- | --- | --- | --- | --- | --- | --- | --- |
|  | Un-adjusted odds ratio | Lower 95% confidence interval | Upper 95% confidence interval | P | Adjusted odds ratio | | Lower 95% confidence interval | Upper 95% confidence interval | P |
| *Cancer type* |  |  |  | P<0.001 |  | |  |  | P<0.001 |
| Bladder | 0.26 | 0.24 | 0.28 |  | 0.24 | | 0.23 | 0.26 |  |
| Brain |  |  |  |  |  | |  |  |  |
| Breast | 0.19 | 0.18 | 0.20 |  | 0.21 | | 0.21 | 0.22 |  |
| Cervix | 0.39 | 0.34 | 0.44 |  | 0.46 | | 0.40 | 0.53 |  |
| Colon | 1.00 | 1.00 | 1.00 |  | 1.00 | | 1.00 | 1.00 |  |
| DCIS | 1.00 | 1.00 | 1.00 |  | 1.00 | | 1.00 | 1.00 |  |
| HL | 0.93 | 0.81 | 1.07 |  | 1.04 | | 0.91 | 1.20 |  |
| Larynx | 0.68 | 0.61 | 0.76 |  | 0.68 | | 0.61 | 0.76 |  |
| Leukaemia |  |  |  |  |  | |  |  |  |
| Liver | 1.69 | 1.53 | 1.86 |  | 1.74 | | 1.57 | 1.91 |  |
| Lung | 2.32 | 2.22 | 2.41 |  | 2.33 | | 2.23 | 2.43 |  |
| Melanoma | 0.08 | 0.07 | 0.08 |  | 0.08 | | 0.07 | 0.09 |  |
| Mesothelioma | 1.79 | 1.55 | 2.06 |  | 1.68 | | 1.46 | 1.94 |  |
| Myeloma | 0.36 | 0.31 | 0.41 |  | 0.36 | | 0.32 | 0.41 |  |
| NHL | 1.55 | 1.47 | 1.64 |  | 1.61 | | 1.52 | 1.70 |  |
| Oesophagus | 2.16 | 2.03 | 2.31 |  | 2.15 | | 2.01 | 2.30 |  |
| Oral | 1.95 | 1.82 | 2.08 |  | 2.07 | | 1.93 | 2.22 |  |
| Other | 1.08 | 1.02 | 1.15 |  | 1.14 | | 1.07 | 1.21 |  |
| Ovary | 1.16 | 1.09 | 1.24 |  | 1.32 | | 1.24 | 1.41 |  |
| Pancreas | 2.75 | 2.57 | 2.94 |  | 2.79 | | 2.61 | 2.99 |  |
| Prostate | 0.57 | 0.55 | 0.59 |  | 0.54 | | 0.52 | 0.56 |  |
| Rectum | 1.00 | 0.95 | 1.05 |  | 1.00 | | 0.95 | 1.05 |  |
| Renal | 0.64 | 0.60 | 0.68 |  | 0.67 | | 0.63 | 0.71 |  |
| Stomach | 1.87 | 1.73 | 2.02 |  | 1.83 | | 1.70 | 1.98 |  |
| Testis | 0.07 | 0.05 | 0.09 |  | 0.08 | | 0.06 | 0.10 |  |
| Thyroid | 0.48 | 0.43 | 0.54 |  | 0.57 | | 0.51 | 0.64 |  |
| Uterus | 0.17 | 0.16 | 0.19 |  | 0.20 | | 0.18 | 0.21 |  |
|  |  |  |  |  |  | |  |  |  |
| Learning disability (Yes) | 1.16 | 0.97 | 1.39 | 0.097 | 1.37 | | 1.12 | 1.67 | 0.002 |
|  |  |  |  |  |  | |  |  |  |
| *Morbidity burden* |  |  |  | P<0.001 |  | |  |  | P<0.001 |
| No morbidity burden | 1.00 | 1.00 | 1.00 |  | 1.00 | | 1.00 | 1.00 |  |
| Low morbidity burden | 0.96 | 0.94 | 0.99 |  | 0.83 | | 0.80 | 0.85 |  |
| Medium morbidity burden | 1.16 | 1.13 | 1.19 |  | 0.80 | | 0.78 | 0.83 |  |
| High morbidity burden | 1.32 | 1.29 | 1.36 |  | 0.71 | | 0.69 | 0.74 |  |
|  |  |  |  |  |  | |  |  |  |
| Sex (Female) | 0.65 | 0.64 | 0.66 | P<0.001 | 0.87 | | 0.85 | 0.90 | P<0.001 |
|  |  |  |  |  |  | |  |  |  |
| *Age* |  |  |  | P<0.001 |  | |  |  | P<0.001 |
| 40 to 44 years | 0.38 | 0.35 | 0.40 |  | 0.64 | | 0.60 | 0.69 |  |
| 45 to 49 years | 0.48 | 0.45 | 0.50 |  | 0.76 | | 0.72 | 0.81 |  |
| 50 to 54 years | 0.67 | 0.65 | 0.70 |  | 0.85 | | 0.81 | 0.89 |  |
| 55 to 59 years | 0.82 | 0.79 | 0.85 |  | 0.88 | | 0.85 | 0.92 |  |
| 60 to 64 years | 0.93 | 0.90 | 0.97 |  | 0.95 | | 0.91 | 0.98 |  |
| 65 to 69 years | 1.00 | 1.00 | 1.00 |  | 1.00 | | 1.00 | 1.00 |  |
| 70 to 74 years | 1.05 | 1.02 | 1.08 |  | 1.04 | | 1.01 | 1.08 |  |
| 75 to 79 years | 1.07 | 1.04 | 1.11 |  | 1.10 | | 1.06 | 1.14 |  |
| 80 to 84 years | 1.16 | 1.12 | 1.20 |  | 1.20 | | 1.15 | 1.25 |  |
| 85 to 89 years | 1.28 | 1.23 | 1.33 |  | 1.38 | | 1.32 | 1.44 |  |
| 90 years and older | 1.28 | 1.21 | 1.35 |  | 1.53 | | 1.42 | 1.63 |  |
|  |  |  |  |  |  | |  |  |  |
| *IMD* |  |  |  |  |  | |  |  | P<0.001 |
| 1 (least deprived | 1.00 | 1.00 | 1.00 | P<0.001 | 1.00 | | 1.00 | 1.00 |  |
| 2 | 1.07 | 1.05 | 1.10 |  | 1.02 | | 0.99 | 1.05 |  |
| 3 | 1.15 | 1.11 | 1.18 |  | 1.05 | | 1.02 | 1.08 |  |
| 4 | 1.26 | 1.22 | 1.30 |  | 1.10 | | 1.06 | 1.13 |  |
| 5 (most deprived) | 1.39 | 1.35 | 1.44 |  | 1.13 | | 1.09 | 1.16 |  |
|  |  |  |  |  |  | |  |  |  |
| Smoking history | 1.53 | 1.50 | 1.56 | P<0.001 | 1.05 | | 1.03 | 1.07 | P<0.001 |
|  |  |  |  |  |  | |  |  |  |
| *Year of diagnosis* |  |  |  | P<0.001 |  | |  |  | 0.066 |
| 2012 | 1.00 | 1.00 | 1.00 |  | 1.00 | | 1.00 | 1.00 |  |
| 2013 | 0.97 | 0.94 | 1.01 |  | 0.99 | | 0.96 | 1.03 |  |
| 2014 | 0.95 | 0.92 | 0.98 |  | 0.98 | | 0.94 | 1.01 |  |
| 2015 | 0.96 | 0.93 | 0.99 |  | 0.98 | | 0.94 | 1.01 |  |
| 2016 | 0.94 | 0.91 | 0.97 |  | 0.96 | | 0.92 | 0.99 |  |
| 2017 | 0.96 | 0.93 | 0.99 |  | 0.97 | | 0.94 | 1.01 |  |
| 2018 | 0.93 | 0.90 | 0.96 |  | 0.95 | | 0.92 | 0.98 |  |

**Table 7. Main effect only model associations between 30-day mortality, patient characteristics, cancer type and learning disability**

|  | **30-day mortality** | | | | |  | | | |
| --- | --- | --- | --- | --- | --- | --- | --- | --- | --- |
|  | Odds ratio | Lower 95% confidence interval | Upper 95% confidence interval | P | Odds ratio | | Lower 95% confidence interval | Upper 95% confidence interval | P |
| *Cancer type* |  |  |  | P<0.001 |  | |  |  | P<0.001 |
| Bladder | 0.64 | 0.57 | 0.71 |  | 0.56 | | 0.50 | 0.63 |  |
| Brain | 1.09 | 0.97 | 1.23 |  | 1.62 | | 1.43 | 1.83 |  |
| Breast | 0.23 | 0.21 | 0.25 |  | 0.29 | | 0.26 | 0.32 |  |
| Cervix | 0.43 | 0.32 | 0.58 |  | 0.73 | | 0.53 | 0.99 |  |
| Colon | 1.00 | 1.00 | 1.00 |  | 1.00 | | 1.00 | 1.00 |  |
| DCIS | 0.01 | 0.00 | 0.03 |  | 0.02 | | 0.00 | 0.06 |  |
| HL | 0.33 | 0.23 | 0.47 |  | 0.56 | | 0.39 | 0.81 |  |
| Larynx | 0.42 | 0.33 | 0.54 |  | 0.53 | | 0.41 | 0.68 |  |
| Leukaemia | 1.38 | 1.26 | 1.51 |  | 1.56 | | 1.42 | 1.71 |  |
| Liver | 2.76 | 2.54 | 2.99 |  | 2.98 | | 2.74 | 3.25 |  |
| Lung | 2.29 | 2.17 | 2.42 |  | 2.32 | | 2.19 | 2.46 |  |
| Melanoma | 0.05 | 0.04 | 0.06 |  | 0.07 | | 0.05 | 0.09 |  |
| Mesothelioma | 1.16 | 1.00 | 1.34 |  | 1.13 | | 0.98 | 1.31 |  |
| Myeloma | 0.70 | 0.62 | 0.79 |  | 0.77 | | 0.68 | 0.87 |  |
| NHL | 0.79 | 0.72 | 0.86 |  | 0.97 | | 0.89 | 1.06 |  |
| Oesophagus | 1.01 | 0.92 | 1.11 |  | 1.07 | | 0.97 | 1.17 |  |
| Oral | 0.22 | 0.19 | 0.26 |  | 0.34 | | 0.28 | 0.40 |  |
| Other | 3.22 | 3.03 | 3.42 |  | 3.49 | | 3.28 | 3.71 |  |
| Ovary | 1.04 | 0.95 | 1.15 |  | 1.50 | | 1.36 | 1.66 |  |
| Pancreas | 3.33 | 3.11 | 3.55 |  | 3.65 | | 3.41 | 3.92 |  |
| Prostate | 0.15 | 0.13 | 0.16 |  | 0.17 | | 0.16 | 0.19 |  |
| Rectum | 0.47 | 0.43 | 0.52 |  | 0.54 | | 0.49 | 0.60 |  |
| Renal | 0.70 | 0.63 | 0.77 |  | 0.88 | | 0.79 | 0.97 |  |
| Stomach | 1.60 | 1.46 | 1.76 |  | 1.54 | | 1.39 | 1.69 |  |
| Testis | 0.09 | 0.04 | 0.21 |  | 0.28 | | 0.13 | 0.61 |  |
| Thyroid | 0.30 | 0.23 | 0.39 |  | 0.61 | | 0.47 | 0.78 |  |
| Uterus | 0.23 | 0.19 | 0.27 |  | 0.33 | | 0.28 | 0.38 |  |
|  |  |  |  |  |  | |  |  |  |
| Learning disability (Yes) | 2.51 | 2.11 | 2.99 | P<0.001 | 3.77 | | 3.10 | 4.59 | P<0.001 |
|  |  |  |  |  |  | |  |  |  |
| *Morbidity burden* |  |  |  | P<0.001 |  | |  |  | P<0.001 |
| No morbidity burden | 1.00 | 1.00 | 1.00 |  | 1.00 | | 1.00 | 1.00 |  |
| Low morbidity burden | 1.07 | 1.01 | 1.13 |  | 0.83 | | 0.78 | 0.88 |  |
| Medium morbidity burden | 1.78 | 1.68 | 1.87 |  | 0.93 | | 0.88 | 0.99 |  |
| High morbidity burden | 3.20 | 3.05 | 3.37 |  | 1.17 | | 1.10 | 1.24 |  |
|  |  |  |  |  |  | |  |  |  |
| Sex (Female) | 1.12 | 1.09 | 1.15 | P<0.001 | 0.93 | | 0.90 | 0.96 | P<0.001 |
|  |  |  |  |  |  | |  |  |  |
| *Age* |  |  |  | P<0.001 |  | |  |  | P<0.001 |
| 40 to 44 years | 0.22 | 0.18 | 0.27 |  | 0.31 | | 0.25 | 0.38 |  |
| 45 to 49 years | 0.32 | 0.28 | 0.37 |  | 0.43 | | 0.38 | 0.50 |  |
| 50 to 54 years | 0.46 | 0.42 | 0.51 |  | 0.55 | | 0.49 | 0.61 |  |
| 55 to 59 years | 0.59 | 0.55 | 0.64 |  | 0.65 | | 0.60 | 0.71 |  |
| 60 to 64 years | 0.81 | 0.76 | 0.87 |  | 0.83 | | 0.78 | 0.89 |  |
| 65 to 69 years | 1.00 | 1.00 | 1.00 |  | 1.00 | | 1.00 | 1.00 |  |
| 70 to 74 years | 1.30 | 1.23 | 1.38 |  | 1.26 | | 1.19 | 1.33 |  |
| 75 to 79 years | 1.61 | 1.52 | 1.70 |  | 1.49 | | 1.41 | 1.58 |  |
| 80 to 84 years | 2.46 | 2.33 | 2.59 |  | 2.15 | | 2.04 | 2.28 |  |
| 85 to 89 years | 3.69 | 3.49 | 3.91 |  | 3.23 | | 3.05 | 3.43 |  |
| 90 years and older | 6.08 | 5.72 | 6.47 |  | 5.79 | | 5.41 | 6.20 |  |
|  |  |  |  |  |  | |  |  |  |
| *IMD* |  |  |  |  |  | |  |  | P<0.001 |
| 1 (least deprived | 1.00 | 1.00 | 1.00 | P<0.001 | 1.00 | | 1.00 | 1.00 |  |
| 2 | 1.20 | 1.15 | 1.25 |  | 1.12 | | 1.07 | 1.18 |  |
| 3 | 1.31 | 1.25 | 1.37 |  | 1.18 | | 1.13 | 1.24 |  |
| 4 | 1.48 | 1.41 | 1.56 |  | 1.29 | | 1.23 | 1.35 |  |
| 5 (most deprived) | 1.69 | 1.61 | 1.77 |  | 1.39 | | 1.33 | 1.46 |  |
|  |  |  |  |  |  | |  |  |  |
| Smoking history | 1.51 | 1.46 | 1.56 | P<0.001 | 1.12 | | 1.08 | 1.16 | P<0.001 |
|  |  |  |  |  |  | |  |  |  |
| *Year of diagnosis* |  |  |  | P<0.001 |  | |  |  | P<0.001 |
| 2012 | 1.00 | 1.00 | 1.00 |  | 1.00 | | 1.00 | 1.00 |  |
| 2013 | 0.94 | 0.90 | 0.99 |  | 0.94 | | 0.89 | 0.99 |  |
| 2014 | 0.84 | 0.80 | 0.89 |  | 0.83 | | 0.79 | 0.88 |  |
| 2015 | 0.79 | 0.76 | 0.83 |  | 0.79 | | 0.75 | 0.83 |  |
| 2016 | 0.73 | 0.70 | 0.77 |  | 0.72 | | 0.69 | 0.76 |  |
| 2017 | 0.71 | 0.67 | 0.74 |  | 0.70 | | 0.67 | 0.74 |  |
| 2018 | 0.67 | 0.64 | 0.71 |  | 0.70 | | 0.66 | 0.74 |  |

### Figures


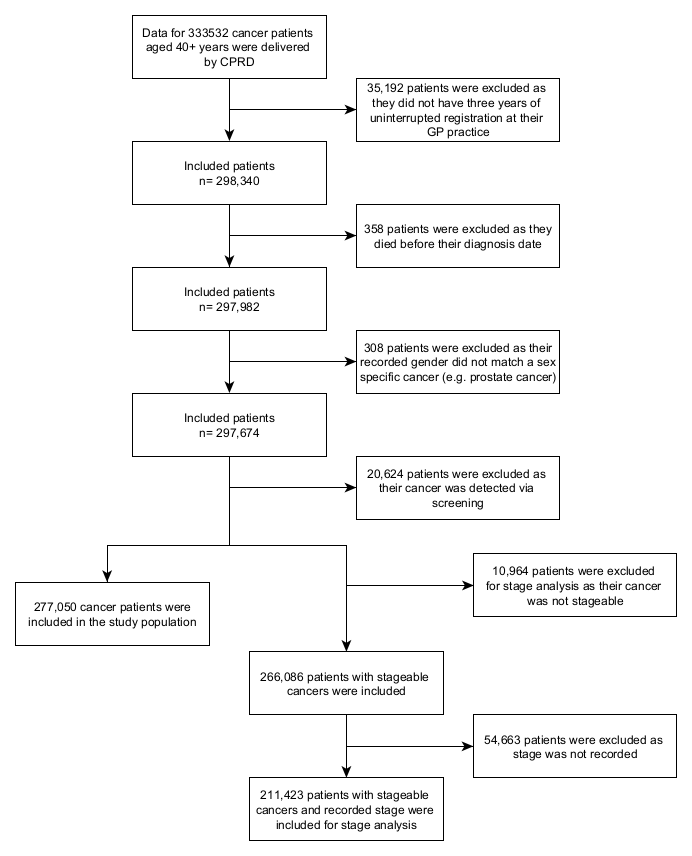


**Figure 1.** Flowchart of the process of selecting the study population. Reprinted from Wiering et al. (2026)[1], used under CC BY 4.0.


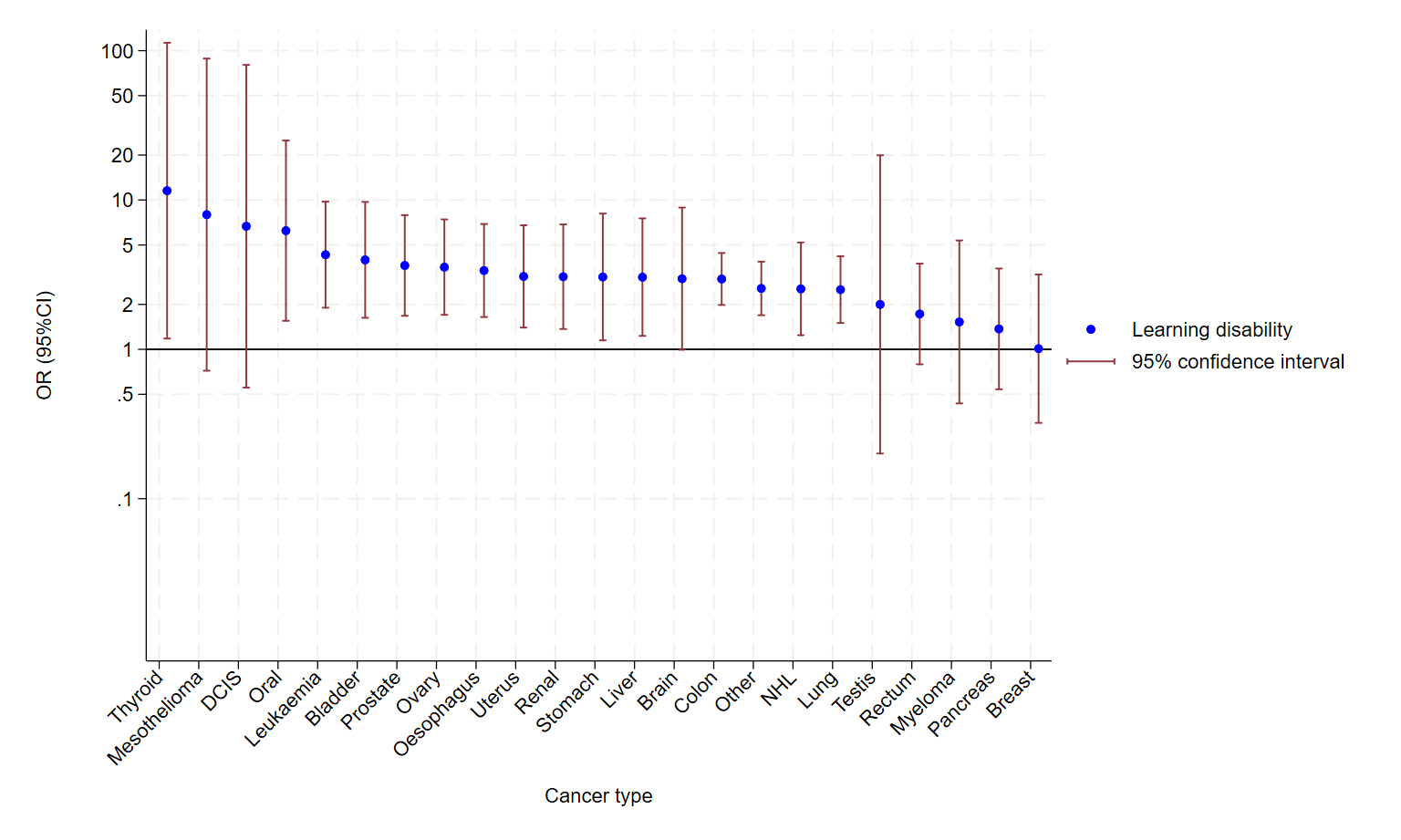


**Figure 2.** Associations between an emergency route to diagnosis and learning disability by cancer type

**
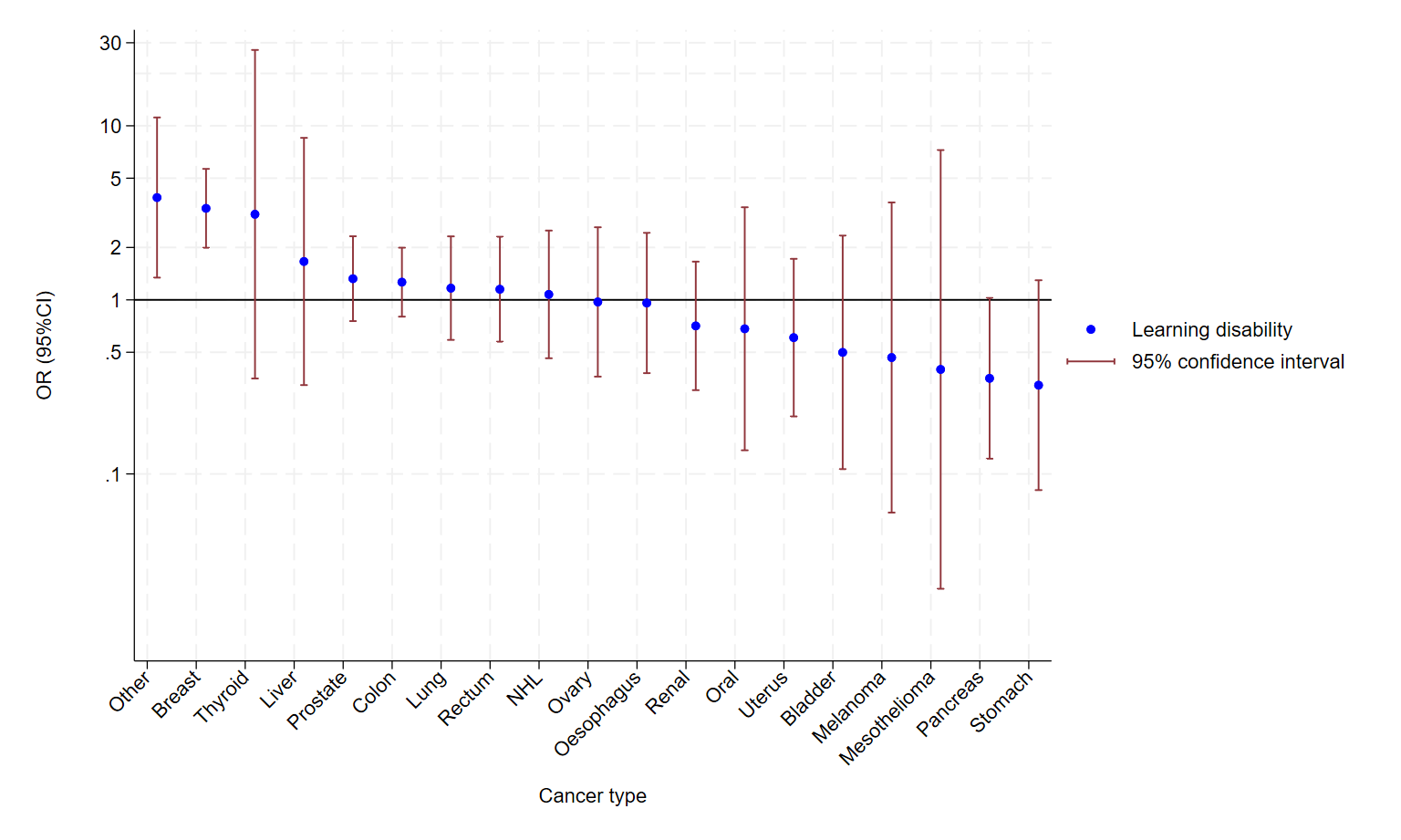

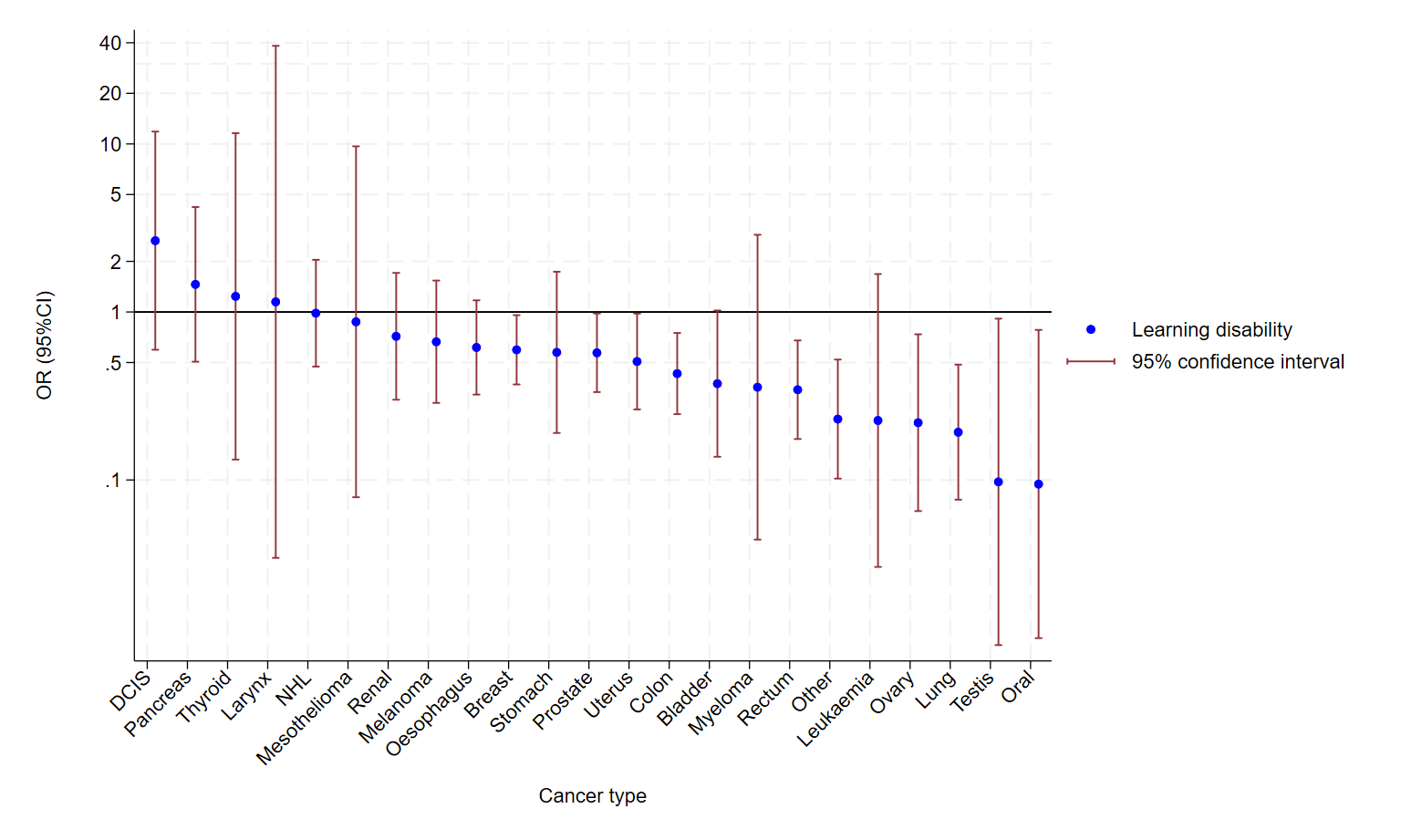
Figure 3.** Associations between an urgent suspected cancer referral (two week wait) route to diagnosis and learning disability by cancer type

**Figure 4.** Associations between cancer stage and learning disability by cancer type

**
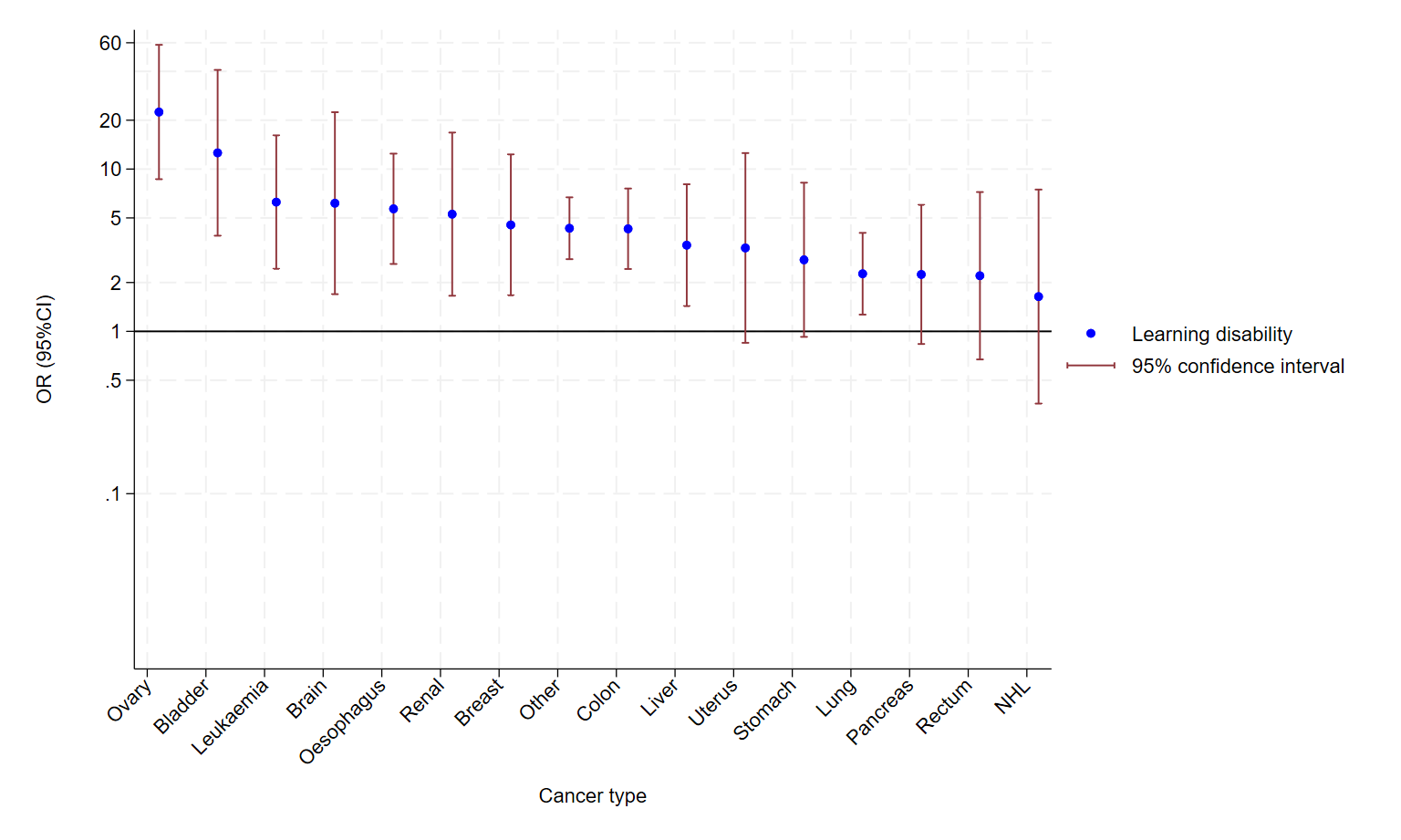
Figure 5.** Associations between 30-day mortality and learning disability by cancer type

**References**

1. Wiering B, Mounce LT, Price SJ, Shotter D, Valderas JM, Merriel SW, et al. The impact of morbidity burden on cancer diagnosis; a retrospective cohort study in England. Cancer Epidemiology. 2026;102:103027.
